# Is Having Urban Green Space in the Neighborhood Enough to Make a Difference? Insights for Healthier City Design

**DOI:** 10.3390/ijerph21070937

**Published:** 2024-07-18

**Authors:** Adriano Bressane, Maria Eduarda Guedes Ferreira, Ana Júlia da Silva Garcia, Líliam César de Castro Medeiros

**Affiliations:** 1 Graduate Program in Civil and Environmental Engineering, São Paulo State University, Bauru 17033-360, Brazil; ajs.garcia@unesp.br; 2 São Paulo State University, Institute of Science and Technology, Environmental Department, São José dos Campos 12209-904, Brazil; meg.ferreira@unesp.br (M.E.G.F.); liliam.medeiros@unesp.br (L.C.d.C.M.)

**Keywords:** nature engagement, urban planning, healthier cities, mental well-being

## Abstract

*Background*: Prior research indicates that engagement with nature is associated with mental well-being; however, the impact of accessibility to urban green spaces (UGS) with suitable infrastructure for visitation and physical activities, like leisure or recreation, remains underexplored, particularly in developing countries. *Purpose*: This study delves into whether merely having green space in the neighborhood is sufficient to impact residents’ mental health in Brazilian metropolitan regions. *Method*: Utilizing a cross-sectional survey, data were collected from 2136 participants. The analyzed variables included the intensity, duration, and frequency of nature engagement, suitability of UGS for visitation and physical activities, and mental well-being indicators measured by the DASS-21 scale. Multivariate statistical analyses and multiple regression models were employed to verify hypothetical relationships. *Results and conclusions*: Higher intensity, duration, and frequency of nature engagement in UGS were significantly associated with lower depression, anxiety, and stress scores. Notably, having urban UGS in the neighborhood alone was not enough to reduce mental health issues. *Practical implications*: The findings point out the need for urban planning policies that prioritize the development of high-quality, accessible green spaces to maximize mental well-being benefits. These insights could inform city designs that foster healthier urban environments. *Future directions*: Longitudinal studies are needed to establish causality between nature engagement and mental health improvements. Further research should incorporate objective measures of nature engagement and explore more aspects of green space quality, such as biodiversity and amenities.

## 1. Introduction

Urbanization has transformed city landscapes, and the expansion of built-up areas often reduces urban green spaces (UGSs), which can play an important role in promoting physical activity, social interactions, and public health. Increasing mental health issues have spurred research into the benefits of UGS [1].

Building on research by Bressane et al. [1,2,3], which found significant associations between nature engagement and reduced mental health issues in Brazilian cities, this study delves into whether simply having urban green space in the neighborhood is enough to significantly impact residents’ mental well-being. Although recognized for their benefits, a deeper understanding of how UGS suitability affects mental well-being is limited. Makram et al. [4] found that neighborhoods with higher NatureScores—an indicator of urban greenness—have significantly lower mental health issues. However, most research overlooks the suitability of green spaces for recreational use, limiting urban planning strategies. Recent findings highlight the multifaceted benefits of green spaces, such as improved sleep and lower blood pressure [5].

Although research in developed nations highlights the mental health benefits of green spaces, similar studies are lacking in developing countries [1,2,3,6,7,8]. The socio-economic and environmental contexts in the Global South differ significantly from those in developed nations, potentially altering the relationship between nature exposure and mental health.

In the context of Brazilian metropolitan regions, the study aims to determine if higher intensity, frequency, and duration of nature activities correlate with lower depression, anxiety, and stress levels, moderated by UGS suitability. The present study hypothesizes that accessibility to UGSs with suitable infrastructure for visitation and physical activities, like leisure or recreation, provides more significant benefits. Higher nature engagement is expected to negatively correlate with mental health issues, moderated by green space suitability. By addressing these aspects, this study aims to deepen the understanding of the role of UGS in promoting mental health and to guide the creation of urban environments conducive to well-being. Confirming the hypothesis could offer important insights for healthier city design.

## 2. Literature Review

### 2.1. Impact of UGS on Mental Health

Research consistently indicates that exposure to UGSs is associated with enhanced psychological well-being. For instance, a study by White et al. [9] utilized a large-scale longitudinal dataset to examine the effects of green space exposure on mental well-being. The findings revealed that individuals residing in areas with greater green space reported higher levels of life satisfaction and lower levels of mental distress compared to those in less green areas. The study underscores the potential of green spaces to foster a sense of well-being among urban residents. A recent study by Helbich et al. [10] further supports this notion, revealing that residential green space is positively associated with lower risks of depression, particularly in women and lower-income groups. This large-scale, cross-sectional study utilized high-resolution satellite imagery to assess green space exposure, providing robust evidence for the psychological benefits of urban greenery.

UGSs also play a crucial role in stress reduction. A systematic review by Bowler et al. [11] analyzed various studies assessing the impact of natural environments on stress. The review found that interaction with green spaces, whether through passive viewing or active engagement, significantly reduced physiological markers of stress, such as cortisol levels, and self-reported stress measures. This body of evidence suggests that urban green spaces can serve as a natural antidote to the high stress levels prevalent in urban settings. A more recent meta-analysis by Twohig-Bennett and Jones [12] confirmed these findings, indicating that exposure to natural environments significantly reduces stress and improves overall mood. This comprehensive review of over 140 studies strengthens the argument for integrating green spaces into urban planning to mitigate stress and enhance mental health.

Beyond general well-being and stress reduction, UGSs have been linked to the alleviation of specific mental health disorders. A notable study by van den Berg et al. [13] explored the relationship between green space and depression. The researchers found that individuals with more access to green spaces were less likely to experience depressive symptoms. This association was particularly pronounced in socioeconomically disadvantaged populations, indicating that green spaces could serve as an equalizer in mental health disparities. Recent evidence from a longitudinal study by Wendelboe-Nelson et al. [14] supports these findings, demonstrating that increased exposure to green space is associated with a lower incidence of major depressive disorder. This study followed participants over a five-year period, providing strong evidence for the long-term mental health benefits of urban greenery.

The mechanisms through which urban green spaces impact mental health are multifaceted. Attention Restoration Theory of Kaplan and Kaplan [15] posits that natural environments facilitate cognitive recovery from mental fatigue, thereby enhancing mental functioning. Additionally, the Biophilia Hypothesis, proposed by Wilson [16], suggests an innate human affinity for nature, which can inherently improve mental health outcomes. These theoretical frameworks provide a basis for understanding the psychological benefits derived from urban green spaces. A recent study by Markevych et al. [17] proposed an integrated conceptual model that combines these theories with empirical findings, suggesting that green spaces impact mental health through multiple pathways, including social cohesion, physical activity, and environmental stress reduction. This model underscores the complex interplay of factors contributing to the mental health benefits of urban green spaces.

### 2.2. Quality and Accessibility of UGS

The quality of UGS encompasses aspects such as maintenance, safety, amenities, biodiversity, and aesthetic value. High-quality green spaces are more likely to be used by residents and provide greater health benefits. Proper maintenance and perceived safety are essential for the utilization of urban green spaces. A study by Van Dillen et al. [18] found that well-maintained and safe green spaces significantly enhance their use and contribute to improved mental health and well-being. The study emphasized that neglect and safety concerns could deter individuals from using these spaces, thereby negating potential health benefits.

The presence of amenities such as playgrounds, benches, walking paths, and sports facilities can significantly enhance the attractiveness and usability of green spaces. Kaczynski and Henderson [19] reported that the availability of diverse recreational facilities in urban parks is positively associated with physical activity levels among residents. This highlights the importance of incorporating various amenities to cater to different user preferences and promote active lifestyles.

Biodiversity and aesthetic appeal also play crucial roles in the perceived quality of green spaces. Fuller et al. [20] demonstrated that higher biodiversity in urban parks is associated with greater psychological benefits, including reduced stress and improved mood. The study suggested that biodiversity enhances the restorative experience of green spaces, making them more beneficial for mental health.

Accessibility refers to the ease with which residents can reach and use green spaces. It is influenced by factors such as proximity, connectivity, and socio-economic barriers. Proximity to green spaces is a key determinant of their use. A study by Schipperijn et al. [21] found that the likelihood of using urban green spaces decreases as the distance from one’s residence increases. This study highlighted the need for equitable distribution of green spaces within urban areas to ensure all residents have easy access. Additionally, the study emphasized the importance of connectivity, such as pedestrian and cycling paths, to facilitate access.

Socio-economic factors can significantly affect access to green spaces. Rigolon [22] reviewed disparities in green space access and found that lower-income and minority communities often have less access to high-quality green spaces. This inequity can exacerbate health disparities, as these communities might not benefit from the mental and physical health advantages provided by green spaces. An inclusive design that considers the needs of diverse populations, including children, the elderly, and people with disabilities, is crucial for enhancing accessibility. A study by Byrne et al. [23] highlighted that inclusive design can promote greater use and enjoyment of green spaces by all community members. The study recommended incorporating features such as accessible paths, sensory gardens, and age-appropriate amenities.

### 2.3. Brazilian Context

A few recent studies have further explored the effect of UGSs on mental well-being in the Brazilian context. Bressane et al. [2] conducted a primary survey in Brazil assessing the association between contact with nature and symptoms of anxiety, stress, and depression. They found that frequent contact with nature significantly reduced the likelihood of these mental health issues, emphasizing the mental health benefits of regular interaction with natural environments. In turn, Bressane et al. [3] found specific patterns and frequencies of nature contact that are most beneficial for mental health, providing insights for urban planning and public health strategies. Bressane et al. [1] investigated how the naturalness of green spaces impacts public well-being. Their study highlighted that more natural green spaces, with higher biodiversity and less human intervention, provided greater mental health benefits, supporting the design of healthier urban environments.

As highlighted in this literature review, while many studies such as those by White et al. [9] and Helbich et al. [10] have established the general benefits of UGSs on mental health in developed countries, there is a notable gap in research within developing countries. This study fills this gap by examining the intensity, frequency, and duration of nature activities and their correlation with mental health outcomes in Brazil, thus providing valuable insights into a different socio-economic and environmental context.

Furthermore, this study addresses the critical but often overlooked moderating role of UGS suitability. The literature review points out that the quality and accessibility of green spaces are crucial for maximizing their mental health benefits [18,19]. By investigating how the quality and infrastructure of green spaces influence mental health benefits, this study offers practical implications for urban planning. Findings from similar contexts, such as the study by Wendelboe-Nelson et al. [14], which highlighted the long-term mental health benefits of green space, support the significance of this research. The use of a diverse sample from Brazilian metropolitan cities further ensures the generalizability of the findings in previous studies conducted in other regions, making this study a valuable addition to the existing body of literature on the benefits of urban green spaces and their role in promoting mental health.

## 3. Materials and Methods

### 3.1. Study Area

This study explores the UGS–mental well-being relationship within metropolitan cities in Brazil, a country with the highest anxiety rate in the world and the third highest rate of depression in Latin America [24]. In Brazil, UGSs are diverse, ranging from public parks to community gardens [25,26]. Rapid urban growth in this country often reduces access to these environments [27]. Brazilian cities, where complex social, economic, and environmental factors shape public health outcomes, face unique challenges in maintaining mental health through green spaces [28,29]. Rapid urbanization and rich biodiversity create a stark contrast, leading to nature disconnection, urban heat island effects, environmental degradation, and adverse mental health impacts [30].

### 3.2. Experimental Design

The survey questionnaire was designed to assess participants’ interaction with nature and their mental health. The variables analyzed include intensity of nature engagement, indicating the physical activity level; duration of nature engagement; frequency of nature engagement; UGS suitability, indicating the accessibility to UGS in the neighborhood with suitable infrastructure for visitation and physical activities; and mental well-being scores, encompassing depression, anxiety, and stress levels. The analysis also controls for sociodemographic factors, including gender, age, income, and education.

### 3.3. Survey Sections

The first section of the survey provided demographic details of participants, including gender, age, income, education, and place of residence. The second section recorded the weekly frequency of engagement with nature, ranging from “less than once” to “more than three times”. The duration of weekly nature interactions was measured in 30 min intervals, from “30 min” to “over 240 min”. In this same section, participants were also asked about the types of activities undertaken during these interactions, categorized by intensity levels: low, moderate, or high. Activities included observing, walking, fishing, meditating, camping, horseback riding, running, cycling, swimming, and others. This categorization was based on physical exertion levels, referencing standards by Haskell et al. [31] and Ainsworth et al. [32], being that low-intensity activities, observing, meditating, walking (minimal physical effort, promoting relaxation and mindfulness); moderate-intensity activities, fishing, camping, horseback riding (moderate physical exertion, more engaging than low-intensity activities); and high-intensity activities, running, cycling, swimming (vigorous, significantly demanding, higher energy expenditure). In the third section, the participants specified whether they have access to UGSs in the neighborhood (within a 300 m radius), with the response options: “yes, with suitable infrastructure for visitation and physical activities”, “yes, but with unsuitable infrastructure for visitation and physical activities”, or “no, there are no UGS in my neighborhood”. The final section included the Depression, Anxiety, and Stress Scale (DASS-21), a validated tool for assessing psychological health [33,34,35,36]. The DASS-21 utilizes a four-point Likert-type scale to gauge symptoms of depression, anxiety, and stress.

### 3.4. Data Collection

Data were collected from a diverse group of respondents in a metropolitan region in Brazil using a dual recruitment strategy. The survey was disseminated via social media and email to educational institutions, leveraging their demographic diversity. Special efforts targeted underrepresented groups, such as older adults and individuals from lower socioeconomic backgrounds, through partnerships with local organizations and public libraries. Reminders were sent to boost participation rates among less active online communities. This strategy aimed to ensure a representative sample of the urban population in Brazil. Previous research has shown the efficacy of using varied channels, including schools and word of mouth, to engage urban populations and enhance participation rates [37]. Collaborations with local organizations and public libraries were specifically chosen to engage underrepresented groups, addressing potential biases and ensuring diverse socioeconomic representation [38].

### 3.5. Data Analysis

Correlations between activity intensity, duration, frequency, and mental health scores were computed, considering the quality of nearby green spaces. This method effectively elucidates how combinations of independent variables (nature contact dimensions) relate to multiple dependent variables (mental health outcomes), providing a comprehensive view of these interactions. Multiple regression analyses determined the impact of activity intensity, duration, and frequency on mental health scores, controlling for sociodemographic variables and green space quality. Interaction effects between green space quality and activity intensity, duration, and frequency on mental health outcomes were also examined. Analyses of variance (ANOVA) compared mean mental health scores across different activity intensity and frequency levels and UGS suitability categories [39]. To ensure the reliability of the scale’s grouping, a robustness check was conducted by calculating Cronbach’s alpha, which resulted in a value of 0.789, indicating satisfactory internal consistency. While a value of 0.8 is traditionally considered good, a Cronbach’s alpha above 0.7 is generally acceptable for most research purposes [40,41,42]. A test power (1 − β) of 0.8 was adopted, indicating an 80% probability of detecting significant effects and minimizing Type II errors [43]. The significance level (α) was set at 0.05, the standard threshold for determining statistical significance while controlling for the false discovery rate. The minimum detectable effect size (rho) was set at 5%.

### 3.6. Ethical Considerations

The study complied with Brazilian ethical standards for human research, as confirmed by approval from the ethical review board (Approval Process #58149622.3.0000.0077). Participant anonymity and confidentiality were maintained throughout the research process.

## 4. Results

This study included a diverse group of 2136 participants, with 59.6% female. Educational attainment was high: 83.5% held a university degree, 16.2% completed high school, and 0.3% had only elementary education. Age distribution was 15.7% young adults (18–25 years), 55.2% adults (26–45 years), and 29.1% middle-aged adults (46–65 years). Income distribution was 16.2% in the lower-income bracket (up to 2 minimum wages), 21.9% in the lower-middle income bracket (2–4 minimum wages), 37% in the middle-income bracket (4–10 minimum wages), and 17.7% in the upper-middle income range (10–20 minimum wages).

Regarding depression, anxiety, and stress scores, stratified by activity intensity, duration, frequency, and UGS suitability, the data revealed that individuals engaging in high-intensity activities (mean depression score = 4.75, SD = ±3.92), for longer durations (mean anxiety score = 2.55, SD = ±3.05), and with greater frequency (mean stress score = 5.32, SD = ±4.17) tend to exhibit lower scores of depression, anxiety, and stress. Notably, those with access to suitable UGSs show lower mental health scores (mean depression = 6.21, anxiety = 2.85, stress = 6.85) compared to individuals without nearby UGSs (mean depression = 6.52, anxiety = 3.27, stress = 6.95) or those with access to inadequate spaces (mean depression = 7.37, anxiety = 2.93, stress = 6.30).

Table 1 and Table 2 present the correlation and multiple regression analysis, respectively, between activity intensity, duration, frequency, and mental health scores, including the quality of green spaces within a 300 m radius.

Significant negative correlations between activity variables (intensity: *r* = −0.261, duration: *r* = −0.221, frequency: *r* = −0.211) and depression scores reinforce the notion that greater nature engagement is associated with better mental well-being. Similar results were found for anxiety (intensity: *r* = −0.120, duration: *r* = −0.157, frequency: *r* = −0.155) and stress (intensity: *r* = −0.176, duration: *r* = −0.176, frequency: *r* = −0.185). The moderate correlation between UGS suitability and mental scores (depression: *r* = −0.107, anxiety: *r* = −0.067, stress: *r* = −0.084) suggests that the quality of nearby UGSs can enhance these benefits.

For depression scores, significant negative coefficients were found for activity intensity (−0.627, *p* < 0.001), duration (−0.098, *p* = 0.002), and frequency (−0.212, *p* = 0.001). Additionally, UGS suitability within the neighborhood also showed a significant negative coefficient (−0.258, *p* = 0.010), suggesting that the quality of nearby green spaces plays an important role in reducing depression. Regarding anxiety scores, significant negative coefficients were also observed for activity intensity (−0.319, *p* = 0.001), duration (−0.094, *p* = 0.006), and frequency (−0.238, *p* = 0.001). UGS suitability also showed a significant negative coefficient (−0.368, *p* = 0.001), reinforcing the importance of high-quality green spaces. Concerning stress scores, as with depression and anxiety, significant negative coefficients were found for activity intensity (−0.278, *p* = 0.005), duration (−0.123, *p* = 0.001), and frequency (−0.213, *p* = 0.003). However, UGS suitability was not significant for stress (−0.166, *p* = 0.500), which may indicate that other factors, beyond this suitability, could moderate the effects of stress.

The apparent discrepancy between low correlations and highly significant regression coefficients may arise from the nature of these statistical analyses. Correlations only measure the strength and direction of a linear relationship between two variables without accounting for the influence of other variables. In contrast, multiple regression analysis considers the simultaneous effects of multiple predictors on the outcome variable, allowing for the control of confounding factors. In our study, while individual correlations between nature engagement variables and mental health scores are moderate, the regression models show significant coefficients. This indicates that when controlling for other variables such as sociodemographic factors and UGS suitability, the combined effect of nature engagement on mental health outcomes becomes more pronounced.

Table 3 presents the results of the ANOVA, conducted to determine if there are significant differences in mean scores of depression, anxiety, and stress among different levels of engagement with nature, stratified by accessibility to suitable UGS within a 300 m radius of the respondents’ residences.

The significant effects (*p* < 0.001) of the interactions suggest that the combination of activity intensity, frequency, and accessibility to quality suitable UGSs has a substantial impact on individuals’ mental well-being.

## 5. Discussion

Our study aimed to explore the relationship between nature engagement (activity intensity, duration, frequency), mental well-being (depression, anxiety, and stress), and accessibility to suitable UGSs. The findings underscore the critical role of UGS suitability in maximizing the mental well-being benefits of nature engagement, corroborating recent studies that have provided further insights into this relationship. For instance, a systematic review by Nguyen et al. [44] highlighted that UGSs contribute significantly to mental health by providing environmental benefits, promoting outdoor activity, and enhancing social cohesion.

Similarly, a study conducted during the post-COVID-19 era emphasized the importance of perceived UGSs for youth mental health, showing a negative correlation between UGSs and anxiety among youth, although the effect on depression was less pronounced [45]. This aligns with our findings that more intense, longer, and frequent activities in high-quality green spaces are associated with lower depression and anxiety scores, as indicated by our multiple regression analysis. Additionally, UGS suitability showed a significant negative coefficient, suggesting that the quality of nearby green spaces plays an important role in reducing depression scores. These results are consistent with the findings of Dadvand et al. [46], who reported that access to green spaces positively impacts general health by reducing mental health issues through increased physical activity and social support. Houlden et al. [47] found that the perceived quality of green spaces was more important than their quantity in predicting mental health outcomes in urban settings. In turn, Van den Berg et al. [48] highlighted that individuals who spent more time in high-quality green spaces reported lower levels of stress and better overall well-being.

Regarding anxiety scores, more intense, longer, and more frequent activities are also associated with lower levels of anxiety. Moreover, UGS suitability showed a significant negative coefficient, reinforcing the importance of high-quality green spaces. These outcomes suggest that urban interventions promoting frequent and intense use of high-quality green spaces can be effective in reducing anxiety among urban residents. This is supported by a review by Liu et al. [49], which found that different types of UGS impact residents’ mental health through various mediators, including stress reduction and emotional stabilization. Marselle et al. [50] also found that the frequency of visits to green spaces was positively associated with lower levels of anxiety and stress, particularly when the spaces were well-maintained and provided opportunities for physical activity.

As with depression and anxiety, significant negative coefficients indicate that more intense, longer, and more frequent activities are associated with lower stress levels. However, UGS suitability was not significant for stress, suggesting that other factors beyond UGS suitability may moderate the effects of stress. These results suggest that promoting physical activities in green spaces can be an effective strategy for reducing stress, but the quality of green spaces may need additional improvements to maximize their benefits. This is echoed in the findings of a recent study that examined the role of UGS suitability on stress, which emphasized the need for well-maintained and accessible green spaces to achieve optimal mental health benefits [6]. Moreover, White et al. [51] also support this finding, suggesting that while green space suitability is important, the specific features and quality of these spaces significantly impact their effectiveness in reducing stress. Their study emphasizes that factors such as the presence of water features, biodiversity, and facilities for physical activities play crucial roles in maximizing the stress-reducing benefits of UGSs. The significant effects revealed by the ANOVA results also emphasize the need for urban policies that promote not only the presence of green spaces but also their suitability, ensuring they are suitable for nature engagement.

These findings confirm the research hypothesis that higher nature engagement is associated with better mental health outcomes and these positive effects are moderated by the quality of nearby green spaces. Therefore, simply having green space in the neighborhood is not enough to make a significant difference in mental well-being outcomes. While the presence of UGS is important, their suitability plays a crucial role in maximizing benefits. Therefore, urban planning policies should focus not only on increasing the quantity of green spaces but also on improving their suitability. Even in an emerging country like Brazil, with high tax rates, Bressane et al. [52] found significant willingness among the population to pay for the maintenance and improvement of UGS, driven by the recognition of health benefits. High-quality green spaces that are accessible, safe, and well-maintained can significantly enhance residents’ mental well-being by promoting more frequent and intense use for recreational and physical activities.

### 5.1. Insights for the Design of Healthy Cities

Table 4 provides key insights for the design of healthy cities based on the study results. Each theme identifies a specific aspect of UGS, highlighting the associated study findings and providing actionable insights for urban planners and policymakers. These insights are aimed at optimizing the design, maintenance, and accessibility of green spaces to enhance the mental health and well-being of city residents. The integration of these recommendations into urban planning strategies can significantly contribute to creating more resilient, inclusive, and health-promoting urban environments. This study has demonstrated that high-intensity activities in green spaces, such as running and cycling, are linked to reduced levels of depression, anxiety, and stress. To promote high-intensity activities, urban planners should consider designing specific areas within green spaces. This can be achieved by allocating certain paths specifically for cycling and others for jogging, with clear signage and mile markers. Additionally, installing outdoor fitness equipment such as pull-up bars, balance beams, and outdoor gyms that cater to various fitness levels allows for circuit training or high-intensity interval training (HIIT) sessions. Hosting regular fitness-related events or classes, such as yoga, or Tai Chi, can attract more visitors and encourage engagement in high-intensity activities.

Our results also showed that the duration and frequency of engagement with nature are also important for mental well-being. To facilitate longer and more frequent interactions with nature, providing comfortable amenities, such as benches, shaded areas, water fountains, restrooms, and Wi-Fi hotspots, can make long stays more comfortable. Educational and engaging signage can encourage visitors to explore different parts of the park, extending the duration of their visits. Scheduling regular educational and recreational programs, such as bird watching tours, nature walks, or art sessions, can also encourage repeated and prolonged visits.

The UGS suitability for various activities was identified as an extremely important factor in moderating the mental health benefits. Designing varied landscapes in green spaces, such as open fields for team sports, quiet wooded areas for meditation or reading, and interactive playgrounds for children, can cater to diverse preferences. Maintaining high standards of upkeep, ensuring pathways are clear, lawns are mowed, and facilities are in good repair, is essential. Increasing the sense of safety with adequate lighting, visible security presence, and emergency call stations enhances usability at different times of the day.

Accessibility to high-quality green spaces is crucial, especially in urban areas where natural spaces are limited. Improving public transportation links to green spaces, providing ample parking, and ensuring pedestrian pathways leading to these areas are safe and well-maintained is fundamental. Involving community members in the planning process helps identify the most needed improvements and preferred features in green spaces. Strategically developing new green spaces in underserved areas ensures balanced accessibility across the city.

The quality of UGSs can significantly enhance the mental health benefits derived from nature activities. Implementing strategies that incorporate a variety of plant species can increase biodiversity, which has been shown to enhance psychological well-being. Including elements like ponds, streams, or fountains can provide calming soundscapes and visual interest, thus increasing the quality of the green space. Integrating environmental art that encourages interaction and engagement provides aesthetic value and promotes mental relaxation.

By focusing on these detailed strategies, urban planners and community leaders can maximize the mental health benefits of green spaces, making them more than just places to relax but vital components of urban public health infrastructure.

### 5.2. Limitations and Future Directions

While our findings underscore the critical role of UGS suitability in maximizing the mental well-being benefits of nature engagement, we acknowledge the potential bidirectional nature of this relationship. It is possible that individuals with better mental health are more likely to engage in outdoor activities and utilize green spaces. This bidirectional relationship is a common challenge in cross-sectional studies, which limits our ability to infer causality definitively. Therefore, we recommend that future research employ longitudinal designs to establish causal links between nature engagement and mental health improvements.

The study did not account for potential confounding variables such as pre-existing mental health conditions, lifestyle factors (e.g., physical activity outside of nature engagement), or environmental stressors (e.g., noise pollution) that could influence mental health outcomes. Future research should control for these factors to isolate the effects of nature engagement more effectively. Furthermore, the study’s focus on depression, anxiety, and stress, as measured by the DASS-21, may overlook other dimensions of mental well-being, such as resilience, social connectedness, and overall life satisfaction. Future studies should incorporate a broader range of mental health indicators.

## 6. Conclusions

This study aimed to elucidate whether the quality of nearby green spaces modulates the mental well-being benefits derived from nature engagement. The findings confirmed the research hypothesis, demonstrating that higher intensity and longer duration of nature activities correlate with reduced scores of depression, anxiety, and stress. Therefore, we conclude that the mere presence of green spaces in urban environments is insufficient to elicit substantial health benefits. Instead, the suitability and quality of these spaces are paramount. Urban planning strategies should prioritize the development and maintenance of high-quality green spaces that are accessible, safe, and conducive to various nature activities. By doing so, urban areas can better support the mental well-being of their residents, promoting healthier and more sustainable city living.

## Figures and Tables

**Table 1:** Association between nature engagement, mental well-being, and UGS suitability.

	Nature Engagement			UGS	Mental Well-Being		
	Intensity	Duration	Frequency	Suitability	Depression	Anxiety	Stress
Intensity	1.000	0.504	0.408	0.112	−0.261	−0.120	−0.176
Duration	0.504	1.000	0.405	0.096	−0.221	−0.157	−0.176
Frequency	0.408	0.405	1.000	0.164	−0.211	−0.155	−0.185
Depression	−0.261	−0.221	−0.211	−0.107	1.000	0.542	0.628
Suitability	0.112	0.096	0.164	1.000	−0.107	−0.067	−0.084
Anxiety	−0.120	−0.157	−0.155	−0.067	0.542	1.000	0.643
Stress	−0.176	−0.176	−0.185	−0.084	0.628	0.643	1.000

**Table 2:** Multiple regression analysis of mental health scores and influencing factors.

	Depression				Anxiety				Stress			
	coef.	*p*	CI 95%		coef.	*p*	CI 95%		coef.	*p*	CI 95%	
			inf	sup			inf	sup			inf	sup
Intensity	−0.627	0.000	−0.801	−0.453	−0.319	0.001	−0.508	−0.131	−0.278	0.005	−0.471	−0.085
Duration	−0.098	0.002	−0.160	−0.037	−0.094	0.006	−0.161	−0.027	−0.123	0.001	−0.193	−0.052
Frequency	−0.212	0.001	−0.339	−0.085	−0.238	0.001	−0.376	−0.100	−0.213	0.003	−0.355	−0.071
Suitability	−0.258	0.010	−0.456	−0.061	−0.368	0.001	−0.582	−0.154	−0.166	0.500	−0.649	0.317
Gender	−0.067	0.680	−0.383	0.250	−0.627	0.000	−0.970	−0.284	−0.643	0.000	−0.987	−0.300
Age	−1.024	0.000	−1.418	−0.630	−1.352	0.000	−1.779	−0.925	−1.355	0.000	−1.783	−0.928
Income	0.721	0.006	0.209	1.233	−0.721	0.006	−1.233	−0.209	−0.734	0.005	−1.246	−0.223
Education	−0.487	0.763	−3.647	2.673	−0.487	0.763	−3.647	2.673	−0.617	0.702	−3.775	2.542

**Table 3:** Differences in mental well-being by nature engagement and suitability of UGS.

	Significance (*p*-Value)		
	Depression	Anxiety	Stress
Intensity	2.0 × 10^−3^	1.45 × 10^−14^	7.54 × 10^−17^
Frequency	1.0 × 10^−2^	9.60 × 10^−2^	1.92 × 10^−1^
Suitability	5.0 × 10^−3^	1.93 × 10^−1^	1.41 × 10^−1^
Intensity × Frequency	2.4 × 10^−2^	6.67 × 10^−1^	8.54 × 10^−1^
Intensity × Suitability	6.8 × 10^−2^	9.36 × 10^−2^	3.94 × 10^−1^
Frequency × Suitability	3.0 × 10^−2^	9.11 × 10^−1^	8.63 × 10^−1^
Intensity × Frequency × Suitability	8.5 × 10^−1^	1.00 × 10^0^	9.99 × 10^−1^

**Table 4:** Insights for the design of healthy cities based on study findings.

Study Findings		Insights for Designing Healthy Cities
Intensity of nature engagement	High-intensity activities in green spaces are associated with lower levels of depression, anxiety, and stress.	Design green spaces that encourage high-intensity activities, such as running and cycling trails, outdoor exercise areas, and sports facilities, to effectively improve residents’ mental health.
Duration and frequency of nature engagement	Longer and more frequent interactions with nature are correlated with better mental health outcomes.	Create green spaces that are inviting for prolonged and frequent visits, with infrastructure supporting long stays, such as benches, picnic areas, and shaded spots, to enhance the duration and frequency of use, promoting greater mental well-being.
UGS suitability for visitation and activities	The quality of green spaces, including their suitability for recreational and physical activities, is crucial for maximizing mental health benefits.	Invest in improving green space infrastructure, ensuring they are well-maintained, safe, and suitable for a variety of recreational activities. This includes well-kept trails, safe play areas, and diverse natural elements such as flower gardens and wooded areas.
Accessibility to high-quality UGSs	Accessibility to high-quality green spaces is significantly associated with reduced symptoms of depression and anxiety.	Plan for the equitable distribution of high-quality green spaces across all city areas, especially in low-income and densely populated neighborhoods, to ensure all residents have equal access to the mental health benefits provided by these spaces.
Interactions moderated by quality of UGSs	The quality of green spaces moderates the positive effects of nature activities on mental health.	Incorporate high-quality elements in UGSs, such as rich biodiversity, presence of water, and leisure facilities, to amplify the benefits of physical and recreational activities in these spaces, leading to more significant improvements in residents’ mental health.

## Data Availability

Ethical restrictions: due to the nature of this research, participants of this study did not agree for their data to be shared publicly, so supporting data are not available.
